# Curative Management of Synchronous Lung and Pancreatic Adenocarcinomas in an Older Patient: A Multidisciplinary Case Report

**DOI:** 10.7759/cureus.79401

**Published:** 2025-02-21

**Authors:** Rita Antunes Santos, Teresa Fraga, Ana Carlota Caetano, Sara Póvoa, Nuno Bonito

**Affiliations:** 1 Medical Oncology, Instituto Português de Oncologia de Coimbra Francisco Gentil, Entidade Pública Empresarial (E.P.E), Coimbra, PRT; 2 Medical Oncology, Instituto Português de Oncologia de Coimbra Francisco Gentil, E.P.E., Coimbra, PRT

**Keywords:** geriatric oncology, lung adenocarcinoma, multidisciplinary oncology, pancreatic adenocarcinoma, synchronous malignancies

## Abstract

Synchronous primary malignancies are uncommon and represent diagnostic and therapeutic challenges, particularly for elderly patients with comorbidities. We report the case of a 78-year-old man with distinct primary adenocarcinomas of the lung and pancreas who was successfully treated with a curative-intent approach. Initially exhibiting unintentional weight loss, asthenia, and gastrointestinal complaints, the initial diagnostic workup, which included computed tomography (CT) and positron emission tomography/computed tomography (PET/CT), revealed a spiculated lesion in the left upper lobe and, incidentally, a hypermetabolic lesion in the pancreatic body. Due to the increased clinical suspicion and the potential for symptomatic progression, the pulmonary lesion was prioritized for further assessment investigation. Bronchial brush cytology indicated non-small cell lung adenocarcinoma, resulting in a left upper lobectomy with lymph node dissection. Histopathology confirmed a 31 mm mixed adenocarcinoma with pleural extension and mediastinal nodal involvement (stage IIIA). After adjuvant chemoradiotherapy, complicated by hematologic toxicity, further evaluation of the pancreatic lesion was conducted. A laparoscopic splenopancreatectomy revealed a 10 mm pancreatic ductal adenocarcinoma from an intraductal papillary mucinous neoplasm (stage IA). The patient underwent six cycles of adjuvant gemcitabine and capecitabine, showing no evidence of recurrence in follow-up imaging. This case features the importance of comprehensive imaging, multidisciplinary collaboration, and personalized treatment in managing synchronous malignancies, particularly considering the treatment approach for elderly patients.

## Introduction

Multiple primary malignancies are defined as two or more distinct cancers occurring in the same individual either synchronously or metachronously. These entities are rare, with reported rates ranging from 1% to 17% [1,2]. The synchronous occurrence of tumors in the lung and pancreas is uncommon and presents complex challenges in diagnosis and management. In elderly patients, additional complications arise from comorbidities and diminished physiological reserve, requiring treatment adjustments and careful patient selection [3-5]. Recent advances in imaging techniques, minimally invasive surgical approaches, and combined-modality therapy have broadened treatment options, even in complex cases. A multidisciplinary team (MDT) approach incorporating thoracic and gastrointestinal surgery, radiology, radiation and medical oncology, and supportive care has become essential for optimizing patient outcomes [1,2,6]. This report outlines the management of synchronous primary lung and pancreatic adenocarcinomas in a 78-year-old patient, demonstrating that curative-intent treatment is feasible through rigorous staging, personalized treatment planning, and coordinated interdisciplinary care.

## Case presentation

The patient was a 78-year-old male who was a retired commercial agent and former light smoker (10 pack-years), with a medical history of type 2 diabetes, dyslipidemia, benign prostatic hyperplasia, and degenerative spinal disease, which involved previous disc herniation surgeries. He presented with more than 10% involuntary weight loss, generalized fatigue, and intermittent gastrointestinal discomfort that had developed over the last two months. His medication regimen included a combination of dapagliflozin/metformin, pitavastatin, tamsulosin, finasteride, and pregabalin. He denied known allergies and maintained an Eastern Cooperative Oncology Group (ECOG) performance status of one. There was no known family history of cancer. The physical examination showed no notable changes.

**Figure 1:**
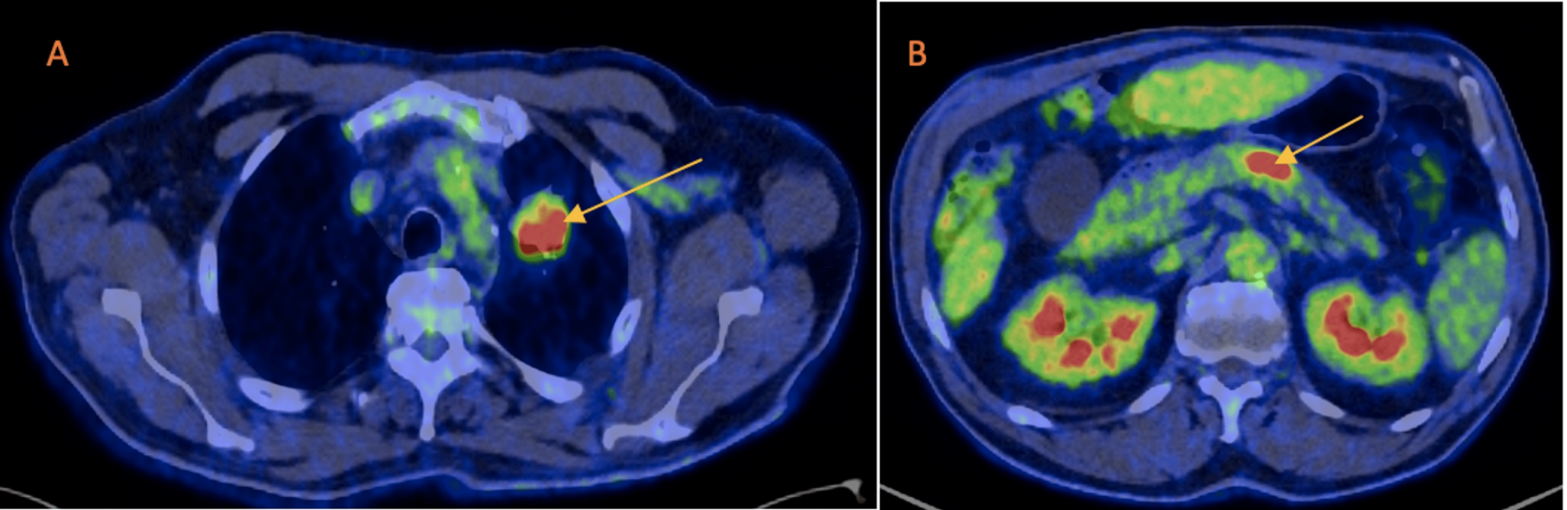
Positron emission tomography/computed tomography (PET/CT) scan using 2-deoxy-2-[fluorine-18]fluoro-D-glucose (18F-FDG) A: The arrow indicates the hypermetabolic characteristics of the lung lesion, which had a maximum standardized uptake value (SUV) of 6.7; B: The arrow indicates focal increased uptake in the pancreatic body, with a maximum SUV of 11.3.

The diagnostic workup began with a contrast-enhanced computed tomography (CT) scan of the chest, which revealed a spiculated mass measuring 30 x 30 x 25 mm in the left upper lobe. This mass exhibited heterogeneous density, microcalcifications, and pleural traction, accompanied by small ipsilateral mediastinal lymph nodes. A subsequent positron emission tomography/computed tomography (PET/CT) scan using 2-deoxy-2-[fluorine-18]fluoro-D-glucose (18F-FDG) confirmed the hypermetabolic characteristics of the lung lesion, which measured approximately 28 x 26 x 31 mm and had a maximum standardized uptake value (SUV) of 6.7 (Figure 1). Additionally, it showed focal increased uptake in the pancreatic body (maximum SUV of 11.3), raising concerns about a potential second primary neoplasm (Figure 1). A cerebral CT scan ruled out brain metastases. Endobronchial ultrasound (EBUS), combined with bronchoscopy evaluation and brush cytology, yielded findings highly suggestive of non-small cell lung adenocarcinoma. The pathological analysis of the biopsied lymph node levels showed no malignant cells. The initial blood test results were normal, with no elevation in tumor markers (including carbohydrate antigen 19-9). The lung adenocarcinoma was subsequently classified clinically as T2a N0 M0, stage IB, according to the eighth edition of the American Joint Committee on Cancer (AJCC).

According to the geriatric assessment, the patient scored 12 points on the G-8 tool, eight points on the Cancer and Aging Research Group (CARG) score, and seven points on the age-adjusted Charlson Comorbidity Index (ACCI). These results indicate a significant level of frailty and a substantial burden of comorbidities, emphasizing the patient's vulnerability to treatment-related toxicity and the critical need for a multidisciplinary approach in tailoring his care therapy.

Following an MDT evaluation involving specialists in thoracic surgery, pulmonology, radiology, radiation oncology, and medical oncology, the patient underwent a left upper lobectomy with mediastinal lymph node dissection through a video-assisted thoracoscopic approach. The procedure was performed without complications. Histopathologic examination of the excised specimen revealed a 31 mm mixed adenocarcinoma exhibiting acinar, papillary, micropapillary, and solid patterns, with evidence of angioinvasion, extension into the visceral pleura, and metastatic involvement of hilar and mediastinal lymph nodes, classifying the tumor as locally advanced, pT2a N2 M0 (stage IIIA, according to the eighth edition AJCC). The multidisciplinary tumor board recommended adjuvant therapy, which comprised the patient receiving weekly carboplatin (area under the curve (AUC) 2) and paclitaxel (45 mg/m²) concurrently with external beam radiotherapy delivered via intensity-modulated helical techniques (total dose 54 Gy in 30 fractions over six weeks to the bronchial stump of the left upper lobectomy and the mediastinal lymph node stations), with daily image guidance. Despite completing the entire six-week course of radiotherapy, the patient received only three of the planned six cycles of chemotherapy due to the development of grade 3 thrombocytopenia, according to the Common Terminology Criteria for Adverse Events (CTCAE), version 5.0. The pancreatic lesion remained stable in staging during the treatment strategy for the lung malignancy.

After recovering from lung-directed treatment, which included hematological toxicities, a subsequent evaluation of the pancreatic lesion using magnetic resonance imaging and endoscopic examination confirmed a localized mass in the body/tail region of the pancreas, with no signs of extrapancreatic spread. Following a new MDT discussion that involved specialists in general surgery, gastroenterology, radiology, radiation oncology, and medical oncology, the patient underwent a laparoscopic splenopancreatectomy (corpo-caudal resection). Histopathological analysis revealed a 10 mm pancreatic ductal adenocarcinoma developing within an intraductal papillary mucinous neoplasm of the pancreatobiliary subtype, with clear resection margins and no lymphovascular or perineural invasion, pT1c N0 M0 (stage IA, according to the eighth edition of AJCC). The proposed adjuvant therapy consisted of a regimen of six cycles of gemcitabine (1000 mg/m² on days one, eight, and 15) and capecitabine (1660 mg/m² in two divided doses, from day one to day 21), administered every 28 days, considering the patient's frailty. The adjuvant therapy was completed with only mild hematologic (grade 2 thrombocytopenia, CTCAE, version 5.0) and gastrointestinal toxicities during the regimen that were solved after a reduction in 20% of the chemotherapy doses.

**Figure 2:**
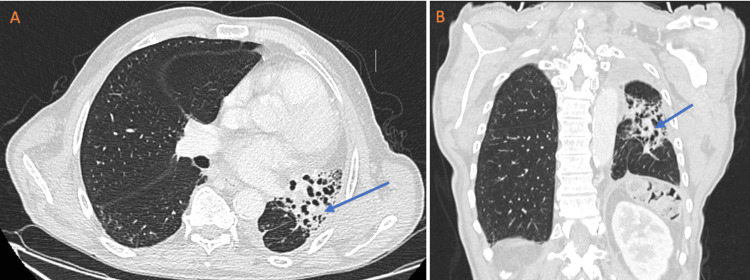
Thoracic computed tomography (CT) scan A: transverse section; B: coronal section Follow-up imaging of the chest revealed stable post-treatment changes, including expected fibrotic alterations (arrow), a reduction in left lung volume, and a slight left pleural effusion.

Follow-up imaging of the chest and abdomen revealed stable postoperative changes, comprising expected fibrotic alterations and a slight left pleural effusion, with no evidence of recurrent or metastatic disease, as can be seen in Figure 2. The patient's performance status remained stable, and he demonstrated improvements in his weight and better management of chronic back pain related to his spinal pathology while remaining free of malignancy.

## Discussion

Managing synchronous primary malignancies in older patients is inherently challenging and requires a multidisciplinary oncologic strategy and a complete geriatric evaluation to guide treatment decisions. A comprehensive geriatric assessment is crucial in these cases, as it evaluates the patient’s physical health and comorbid conditions, cognitive function, nutritional status, and social support systems. These evaluations help adjust treatment intensity, anticipate potential complications, and optimize supportive care measures [5,7].

The patient’s frailty was quantitatively evaluated using the G-8 screening tool (12 points) and the CARG score (eight points), indicating an intermediate risk of grade 3 or higher treatment-related toxicity, and the ACCI (seven points). These findings indicate a significant level of frailty and a considerable comorbidity burden, both of which are linked to an increased risk of treatment-related toxicity and poorer outcomes [7-9]. Nonetheless, the patient’s sustained good performance status and thorough preoperative assessment enabled the implementation of an aggressive yet personalized therapeutic strategy. This strategy prioritized the resection of the higher-stage lung adenocarcinoma, followed by adjuvant chemoradiotherapy (with adjustments to manage hematologic toxicity), and subsequently addressed the pancreatic lesion using minimally invasive surgery and adjuvant chemotherapy.

Recent guidelines emphasize that treatment choices for older cancer patients should not depend solely on age; they must also include thorough geriatric assessments to identify vulnerabilities and guide individualized therapy [7-10]. Our case demonstrates that with careful patient selection and well-coordinated multidisciplinary care, even elderly individuals with significant frailty and multiple health issues can tolerate and benefit from curative-intent treatments. The successful completion of lung and pancreatic-directed treatments in this patient justifies the necessity of integrating geriatric principles into cancer management, ultimately resulting in positive outcomes without compromising quality of life. Additionally, histopathologic analysis also revealed that both neoplasms exhibited features indicative of glandular differentiation. This highlights the need for further molecular studies to evaluate potential underlying genetic factors and predispositions.

## Conclusions

This case report demonstrates that synchronous lung and pancreatic adenocarcinomas can be successfully treated with a curative approach in selected elderly patients. By combining extensive imaging, targeted surgical resection, and adapted adjuvant therapies within a multidisciplinary setting, positive outcomes can be achieved despite the challenges of advanced age and comorbidities. This approach underlines the crucial role of coordinated care in optimizing treatment sequencing and surveillance, ensuring that elderly patients obtain optimal oncologic management.
